# The Transcription Factor Rbf1 Is the Master Regulator for *b*-Mating Type Controlled Pathogenic Development in *Ustilago maydis*

**DOI:** 10.1371/journal.ppat.1001035

**Published:** 2010-08-05

**Authors:** Kai Heimel, Mario Scherer, Miroslav Vranes, Ramon Wahl, Chetsada Pothiratana, David Schuler, Volker Vincon, Florian Finkernagel, Ignacio Flor-Parra, Jörg Kämper

**Affiliations:** 1 Karlsruhe Institute of Technology, Institute for Applied Biosciences, Department of Genetics, Karlsruhe, Germany; 2 MPI for Terrestrial Microbiology, Department for Organismic Interactions, Marburg, Germany; 3 Qiagen GmbH, Hilden, Germany; 4 Kasetsart University, Department of Microbiology, Bangkok, Thailand; 5 Institute for Molecular Biology and Tumor Research, Marburg, Germany; 6 Centro Andaluz de Biología del Desarrollo, Universidad Pablo de Olavide-Consejo Superior de Investigaciones Científicas, Sevilla, Spain; University of Wisconsin-Madison, United States of America

## Abstract

In the phytopathogenic basidiomycete *Ustilago maydis*, sexual and pathogenic development are tightly connected and controlled by the heterodimeric bE/bW transcription factor complex encoded by the *b*-mating type locus. The formation of the active bE/bW heterodimer leads to the formation of filaments, induces a G2 cell cycle arrest, and triggers pathogenicity. Here, we identify a set of 345 bE/bW responsive genes which show altered expression during these developmental changes; several of these genes are associated with cell cycle coordination, morphogenesis and pathogenicity. 90% of the genes that show altered expression upon bE/bW-activation require the zinc finger transcription factor Rbf1, one of the few factors directly regulated by the bE/bW heterodimer. Rbf1 is a novel master regulator in a multilayered network of transcription factors that facilitates the complex regulatory traits of sexual and pathogenic development.

## Author Summary

The basidiomycetous fungus *Ustilago maydis* is the causal agent of the smut disease on corn. The fungus exhibits two different life-styles, a saprophytic and a pathogenic stage, where it grows yeast-like by budding, or as filamentous, dikaryotic hyphae, respectively. The switch between these two stages is controlled by a heterodimeric transcription factor, bE/bW, which is encoded by the *b*-mating type locus. We have now identified the genes that are regulated in response to the activation of bE/bW, following the *b*-mediated developmental change, and address their contribution to the altered morphology and pathogenic development. Interestingly, most of the *b*-responsive genes are not regulated directly by the bE/bW proteins, but require the action of a second transcription factor, Rbf1, which is induced by bE/bW. Rbf1 defines a novel master regulator as a central component in a multilayered network of different, hierarchically ordered transcription factors that facilitate the complex regulatory traits to coordinate morphology as well as sexual and pathogenic development.

## Introduction

In a wide range of fungi, complex developmental traits such as cell identity, morphogenesis and sexual development are controlled by mating type loci [1], [2], [3]. In the smut fungi, a group of plant pathogens, these traits also include the ability to infect their host plants. In *Ustilago maydis*, a smut fungus that infects maize, it is the *b*-mating type locus that is critical for both sexual as well as for pathogenic development. Similar to other smuts, *U. maydis* exhibits a dimorphic life cycle. The haploid, cigar-shaped cells, called sporidia, multiply by yeast-like budding, and the dikaryon, which is formed upon the fusion of two compatible sporidia, grows as a filament. This switch in cell morphology is accompanied by an alteration of the life-style. While the sporidia are apathogenic and grow strictly saprophytic, the filament is biotrophic, i.e. it depends on the living tissue of its host plant maize for further development. Initially, the dikaryotic hypha consists of a long tip cell with the accumulated cytoplasm; the succeeding, older parts consist of “empty” cells that are separated by regularly spaced septae. Cell division is stalled until the hypha has penetrated the cuticula of a corn plant, and only then a “true” filament with multiple septated compartments is formed. Upon plant invasion, hyphae traverse the plant without harming the cells and without an apparent host defense response. After several days, the fungus induces plant tumors, coinciding with a massive proliferation of fungal hyphae [for review, see 4].

In order to fuse and to form the pathogenic filament, the two sporidia must carry different alleles both of the biallelic *a*- and of the multiallelic *b*-mating type locus. The *a*-locus encodes a pheromone/receptor system required for cell sensing, initiation of filamentous conjugation tubes, and cell fusion. After fusion, the crucial step for the initiation of the pathogenic phase is the formation of a heterodimeric complex of two homeodomain proteins, bE and bW, which are encoded by the *b*-mating type. This bE/bW complex is formed only when the two proteins are derived from different *b*-alleles, and is sufficient to initiate the switch from budding to filamentous growth. Concomitantly, activation of *b* leads to a cell cycle arrest that is only released after host plant infection. It has been shown conclusively that the bE/bW complex is sufficient to initiate the pathogenic development, as exemplified by haploid “solopathogenic” strains that harbor different alleles of *bE* and *bW* and that are capable to infect plants without a mating partner [5]. Thus, it is conceivable that genes regulated by the bE/bW heterodimer are involved in (1) the establishment of the biotrophic phase, (2) cell cycle regulation and (3) the dimorphic transition from budding to the polarized growth of the filament. However, until now, only four *b*-regulated genes have been identified with impact on these processes, three of which are required during the very early infection stages. *biz1* encodes a zinc finger transcription factor that is involved in the G2 cell cycle arrest preceding plant penetration as well as in the induction of appressoria, specific infection structures at the tip of penetrating hyphae [6]. The mitogen-activated protein (MAP) kinase Kpp6 is required for the subsequent step: *U. maydis* strains harboring a non-activatable *kpp6* allele still form appressoria, but are defective in the penetration of the plant cuticula [7]. After plant penetration, the *clp1* gene is required for further proliferation of dikaryotic filaments *in planta*. *clp1* mutant strains still penetrate the plant cuticula, but development is stalled prior the first mitotic division; in addition, mutant strains do not form clamps, a structure that ensures the proper distribution of nuclei in the dikaryotic hyphae [8]. Interestingly, the induced expression of *clp1* strongly interferes with the *b*-dependent induction of several of the genes regulated by the bE/bW-heterodimer, indicating that Clp1 may modulate the activity of the bE/bW complex. And finally, the *b*-dependently expressed cyclin Pcl12 is involved in the polarized growth of the *b*-dependent filament, but is dispensable for pathogenic development [9].

The bE/bW heterodimer binds to a conserved sequence motif, the *b*-binding sequence (*bbs*) that has been identified in the *b*-dependently induced *lga2*-gene [10]. Out of the 20 *b*-dependent genes identified so far, only two additional genes were found to harbor the *bbs*-motif: the above mentioned *clp1* gene, and *frb52*, a gene with unknown function [11]. As the majority of *b*-controlled genes is obviously not directly regulated by bE/bW, it appears likely that the bE/bW heterodimer triggers a regulatory cascade with a limited number of direct targets genes. Thus, these “class I” genes should encompass regulators that trigger the regulation of the larger number of indirect, “class II” *b* targets. It was proposed that these regulators play pivotal roles either in all (as master regulator) or distinct (as relay) *b*-dependent processes.

Here, we employed *U. maydis* strains that harbor inducible combinations of the *bE* and *bW* genes [11] and DNA array technology to investigate the *b*-dependent processes in a time-resolved manner. Our analysis provides insight in the complex interconnection of cell cycle regulation during the dimorphic switch and highlights the specific characteristics of the “pathogenic” hyphae. Most important, we identify the zinc-finger transcription factor Rbf1 as a novel master regulator that is required for all *b*-dependent processes.

## Results

### Genes regulated by the bE/bW heterodimer are involved in cell cycle regulation, cell wall remodeling and secretion of effector candidates

**Figure 1:**
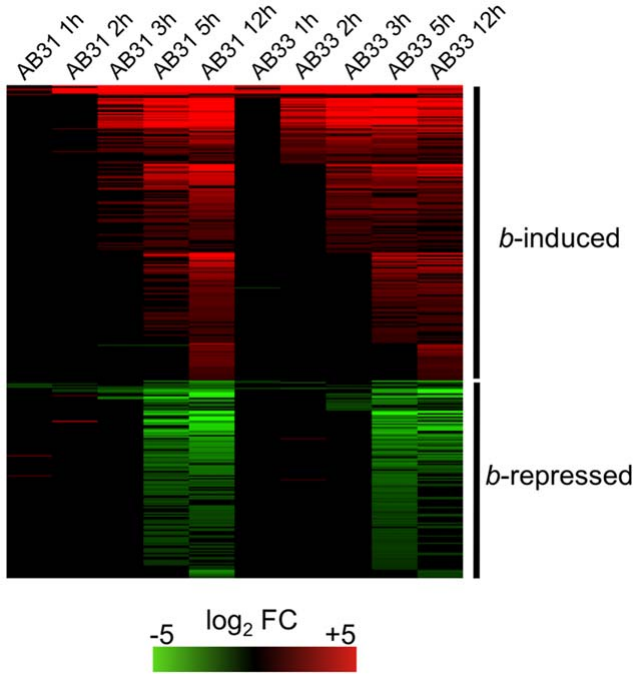
*b*-dependent gene expression. In *U. maydis* strains AB31 and AB33, expression of the *bE1* and *bW2* genes is controlled via the arabinose-inducible *crg1*-promoter and the nitrate-inducible *nar1*-promoter, respectively. Strains AB32 and AB34 are isogenic to AB31 and AB33, respectively, but harbor the incompatible *bE2* and *bW2* genes as control. *bE/bW* genes were induced by shifting the cells to array medium containing arabinose (AB31 and AB32) or nitrate (AB33 and AB34). Cells were harvested at the given time points, RNA was extracted and used for microarry-analysis (Affymetrix MPIUstilagoA gene chip). The heat-map indicates the significant changes (adjusted P<0.01; change >2) in gene expression for AB31 and AB33 at a given time point relative to the control strains AB32 and AB34. Order of genes from top to bottom and expression changes are given in Suppl. Table S1. Details for the statistics and filters applied are given in “Material and Methods”.

In order to identify genes regulated by the bE/bW heterodimer, we performed microarray experiments with custom Affymetrix arrays (MPIUstilagoA) covering 5823 of the predicted 6786 *U. maydis* genes. Changes in gene expression were monitored during a 12-h time course (with samples taken at 1h, 2h, 3h, 5h, 12h) using the haploid *U. maydis* strains AB31 and AB33 that harbor the *bE1* and *bW2* genes under the control of the arabinose-inducible *crg1* promoter and the nitrate-inducible *nar1* promoter, respectively [11]. Induction of *bE1*/*bW2* in these strains results in a filament that resembles the infectious hypha formed after fusion of compatible sporidia [11]. Strains AB32 and AB34, which harbor the incompatible *bE2* and *bW2* combination, were used as controls. Expression of *bE* and *bW* genes was induced by a shift from glucose- to arabinose (AB31 and AB32) or from glutamine- to nitrate containing media (AB33 and AB34). The expression profiles after b-induction in AB31 and AB33 were similar, but not identical. Firstly, the use of different media had an effect on gene expression, and, secondly, the use of the *crg1* promoter resulted in gene expression values that were two- to fivefold higher when compared with *nar1*-driven gene expression (Suppl. Fig. S1). To account for expression changes caused by the medium shift, we considered changes only as relevant when the expression for a particular gene was altered significantly in both AB31 and AB33 in at least one time point (change in expression ≥2, adjusted p-value ≤0.01). Using these criteria, 206 genes were induced and 139 were repressed in response to *b*-induction (Fig. 1; Suppl. Table S1). Within this list, all genes with a significant *b*-dependent regulation identified in previous studies were present, emphasizing the validity of the global approach and the quality of our data set (Suppl. Table S2).

From the 345 *b*-regulated genes, a total of 239 were functionally classified using the Blast2Go tool [12]. Using enrichment analysis, we did not observe a significant over-representation of *b*-induced genes in any of the Gene Ontology (GO) categories (http://www.geneontology.org). However, for the *b*-down-regulated genes, we observed a significant enrichment of the GO categories “Cell Cycle” (GO:0007049; 29 genes), “Chromosome” (GO:0005694; 25 genes) and “DNA metabolic process” (GO:0006259; 19 genes), “Cytoskeleton” (GO0005856; 16 genes) and “Microtuble cytoskeleton” (GO:0015630; 9 genes) (Suppl. Table S3).

The induction of the active bE1/bW2-heterodimer leads to a G2 cell cycle arrest, and in accordance with this observation we found *cln1*, *clb1* and *clb2*, which encode a G1-type cyclin and two B-type cyclins [13], [14] among the down-regulated genes (−29.9-fold, −7.7-fold and −2.6-fold, respectively; Suppl. Table S1). *cln1* and *clb1* are involved in G1 to S transition, *clb1* and *clb2* in the G2 to M transition; thus, it is expected that these genes are poorly expressed in cells that are arrested in G2. For *clb1* it has been shown previously that the repression leads to a G2 cell cycle arrest [14]; thus, the observed low expression of this cyclin may trigger the *b*-induced G2 arrest. Additionally, we find *um03928*, encoding a homologue of the *S. pombe* Cdr2 protein, as 40.7 fold down-regulated. In *S. pombe*, Cdr2 functions as a mitotic inducer via the Wee1 kinase and is required for G2/M transition [15]. In *U. maydis*, Wee1 has been shown to be a central regulator for G2/M transition [16]; however, a function for the Cdr2 homologue *um03928* has not been assigned yet. Another level of complexity may be achieved via the up-regulation of the Cdk5 associated Pho80 Cyclin Like protein Pcl12 (49.1-fold, Suppl. Table S1). Induced expression of *pcl12* leads to a G2 cell cycle arrest, and promotes filamentous growth [C. Pothiratana and J. Kämper, unpublished; 9]. Thus, the *b*-induced cell cycle arrest may be realized via the synchronized regulation of independent pathways. In line with the cell cycle arrest, we observe the repression of genes involved in DNA-replication and nucleotide metabolism, as, for example, *um01008*, encoding the catalytic subunit of DNA polymerase epsilon (3.6-fold down-regulated at 12h), or *um06402*, encoding a DNA replication licensing factor (3,2-fold down-regulated at 12 h; Suppl. Table S1, FunCat DNA).

Several of the *b*-regulated genes can be attributed to the morphological switch from budding- to filamentous growth. A total of 20 genes with a potential function in cell wall synthesis or modification was found to be induced, starting 3 h after *b*-induction, which coincides with the onset of filamentation; five additional genes were repressed (Suppl. Table S1, FunCat: CW). These genes encode for chitin synthases as well as for exochitinases, chitin deacetylases, and exo- and endoglucanases, indicating that the cell wall composition is altered during the switch from sporidia to hyphae.

The largest “functional” group (74 genes) encodes for potentially secreted proteins. 34 of them have no ascribed function, and of these 15 are specific for *U. maydis*. Such secreted proteins are candidates for effectors that may play a role in the establishment of the biotrophic interaction (Suppl. Table S1, secreted).

### The bE/bW heterodimer regulates the expression of transcription factors required for pathogenic development

To identify *b*-dependent genes important for pathogenic development, we focused initially on genes whose expression was “strictly” dependent on the presence of the bE/bW heterodimer, i.e. genes that showed only basal expression levels in strains AB32 and AB34 and showed a more than 10-fold induction upon expression of an active bE1/bW2-heterodimer. None of the 53 genes that fulfilled these criteria showed a significant similarity to known pathogenicity factors. Potential exceptions were *dik6* and *dkh6*, which encode two related seven trans-membrane (7TM) domain proteins. 7TM proteins represent an extended protein family in *M. grisea* that is discussed to function in plant/pathogen interactions [17]. However, neither the single, nor the double deletion of the two genes had an impact on pathogenic development (Suppl. Table S1, G. Weinzierl and J. Kämper, unpublished). In total, we deleted 30 of the 53 strictly *b*-dependent genes in the haploid, solopathogenic strain SG200; in addition, nine genes have been analyzed in the course of previous studies. 35 of the 39 deletion strains did not show altered virulence when assayed in plant infection assays.

However, the individual deletion of each of the five genes encoding proteins with potential regulatory functions affected pathogenic development or filamentous growth (Suppl. Table S1). Among these genes was *clp1 (um02438)*, which has been identified in the course of this study and has been shown to be required for pathogenic development and *in planta* proliferation [8]. The *biz1* gene (*um02549*) encodes a C2H2 zinc finger transcription factor that is required for pathogenic development and efficient appressoria formation [6]. In addition, we could show that the deletion of two genes encoding potential homeodomain transcription factors (*um12024* and *um04928*, termed *hdp1* and *hdp2*) impaired filamentous growth or led to loss of pathogenicity, respectively; the detailed characterization of these two genes will be published elsewhere. Here we will focus on the analysis of *um03172*, encoding a potential C2H2 zinc finger transcription factor.

### The b-dependently induced *rbf1* gene encodes a zinc-finger transcription factor

**Figure 2:**
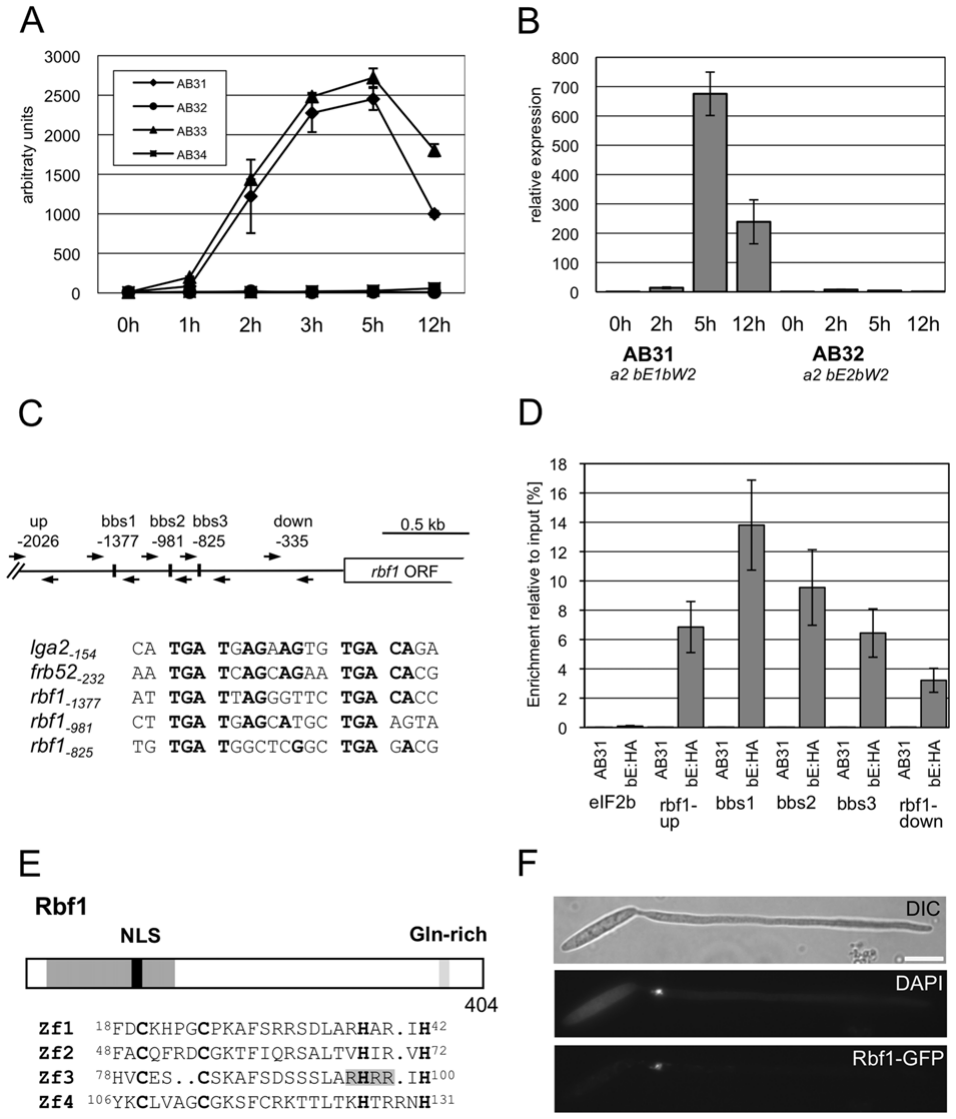
Structure and expression of Rbf1. (*A*) Microarray analysis of *b*-dependent *rbf1* expression after induction of compatible (AB31 and AB33) and incompatible (AB32 and AB34) combinations of *bE* and *bW*. Shown are the mean expression values of two biological replicates and the standard deviation (SD) (*B*) qRT-PCR analysis of *rbf1* expression after induction of compatible (AB31) and incompatible (AB32) combinations of *bE* and *bW*. Samples were taken at the time-points indicated. qRT-PCR analysis was performed using the constitutively expressed *ppi* gene (*um03726*) for normalization. Expression was calculated relative to the lowest expression value. Shown are mean values of two technical replicates. (*C*) Overview of primer binding sites in the *rbf1*-promoter used for qChIP experiments and alignment of three putative b-binding sites (*bbs*) in the *rbf1* promoter region to the *bbs* of *lga2* and *frb52* [10], [11]. Nucleotide positions indicated are relative to the start codon. Nucleotides identical to the *bbs* in *lga2* [10] and *frb52* [11] are in bold. (*D*) qChIP analysis of bE1 binding to the *rbf1*-promoter in strains AB31 and AB31bE1:3xHA 5h after induction of the bE1/bW2-heterodimer. AB31bE1:3xHA harbours a HA-tagged bE1 protein used for immunoprecipitation with anti-HA-antibody. Numbers give the enrichment in % of the input-DNA of the PCR amplicons in DNA co-immunoprecipitated with HA-antibody. No significant enrichment was observed in control strain AB31. In AB31bE1:3xHA, the PCR-amplicon spanning bbs1 (bbs^−1377^) is significantly enriched (t-test) when compared to the amplicon spanning a control region (−2026) (p = 5.71 10^−5^). As additional control, a region from the *eIF2b* gene (*um04869*) was used. Given are the mean values of three technical replicates of three independent experiments each, and the standard deviation (SD). (*E*) Structure of the Rbf1 protein. The potential C2H2 zinc finger domain (aa 18 to 131) and a putative NLS (RHRR) (aa 95 to 98) within this domain are marked in dark grey and black, a glutamine rich sequence (aa 365 to 373) is marked in grey. The alignment shows the four C2H2 zinc finger domains; the conserved cysteine and histidine residues are in bold. (*F*) Subcellular localization of the Rbf1-3xeGFP fusion protein. Strain AB31*rbf1:3eGFP* (UMS63) was induced in CM medium supplemented with 1% arabinose (CMA) for eight hours. The functional Rbf1-3xeGFP fusion protein localizes to the nucleus. Cells were stained with DAPI to visualize nuclei. Scale bar corresponds to 10 µm.

**Figure 3:**
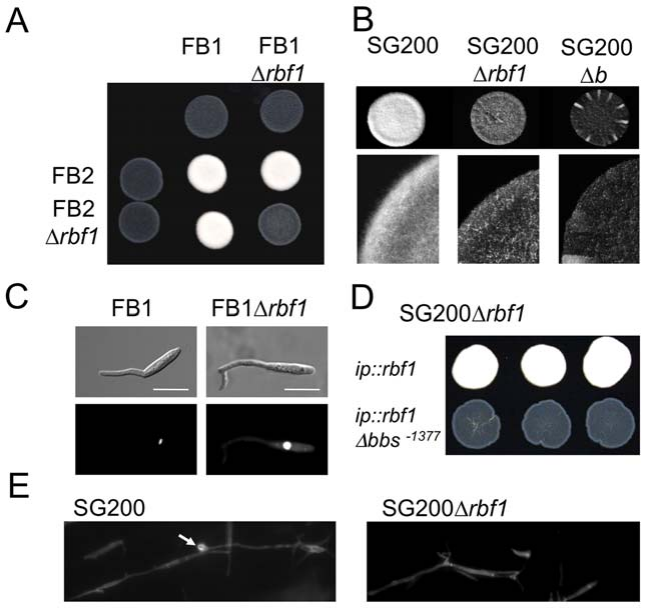
Rbf1 is required for *b*-dependent filament formation. (*A*) Mixtures of wild-type *U. maydis* strains FB1 (*a1b1*) and FB2 (*a2b2*) and of the respective Δ*rbf1* strains and (*B*) of the solopathogenic strain SG200 (*a1mfa2 bE1bW2*) and its *Δrbf1* and *Δb* derivatives were spotted on PD charcoal plates to induce filament formation. Filamentation is drastically reduced in all Δ*rbf1*-strains. (*C*) FB1 and FB1Δ*rbf1* cells grown in CM medium supplemented with 1% glucose (CMD) (OD_600_ = 0.2) were treated with synthetic a2 pheromone (2.5 µg/ml) for six hours; nuclei were visualized by DAPI staining. Rbf1 is not required for the pheromone-induced conjugation tube formation and cell cycle arrest. Scale bar = 10 µm. (*D*) Transformants of SG200Δ*rbf1* obtained by integration into the *ip*-locus of (1) the *rbf1* open reading frame with the wild-type 3 kb 5′-upstream region (*ip::rbf1*), or (2) the *rbf1* open reading frame with a 3 kb 5′-fragment harbouring a deletion of the *bbs*-motif at position −1377 (*ip::rbf1Δbbs^−1377^*). Shown are three independent transformants each spotted on PD charcoal plates. While the *rbf1* gene with the wt-promoter fragment is capable to complement the *rbf1* deletion, restoring filamentous growth, the deletion of *bbs^−1377^* renders the transforming fragment inactive, indicating that binding of the bE1/bW2-heterodimer to *bbs^−1377^* is required for *rbf1* expression. (*E*) Calcofluor staining of fungal hyphae on the leaf surface. 16 hours post inoculation, both *U. maydis* SG200 and SG200Δ*rbf1* have formed filaments; no appressoria were found in SG200Δ*rbf1*. The arrow marks the appressorium.

Due to the initially observed phenotype (see below), the *U. maydis* gene *um03172* was termed *rbf1* (regulator of *b*-filament). According to our microarray analysis, *rbf1* expression was strongly induced early after *b*-induction (Fig. 2A). Significant expression was detected already 1h after *b*-induction in AB33 (13.6-fold induction), and expression peaked at 2h to 3 h (176.3-fold in AB33 at 2h and 297.4-fold in AB31 at 3 h, respectively; Fig. 2A). In the control strains AB32 and AB34 *rbf1* expression was not detectable. The *b*-dependent expression of *rbf1* was confirmed by qRT-PCR using strains AB31 and AB32 (Fig. 2B). Within the *rbf1* promoter, we identified three motifs with similarities to the previously identified b-binding sequences (*bbs*) (Fig. 2C). We used an AB31 derivative expressing the bE1 protein fused to a triple HA-tag (AB31bE1:3xHA) for quantitative chromatin immunoprecipitation analysis (qChIP). Induction of *bE1:3xHA/bW2* genes in this strain led to filamentous growth (see below), demonstrating that the bE1:3HA protein is functional (data not shown). In a qChIP analysis with bE1:3xHA, a significant enrichment (P = 5.7 10^−5^, Students t-test) was observed for the PCR amplicon covering the *bbs*-motif located at position −1377, when compared to a amplicon covering a region further upstream in the *rbf1* promoter (Fig. 2D). The *bbs* ^−1377^-motif shares also the highest sequence similarity with the previously described *bbs*-motifs (Fig. 2C). When the *rbf1* gene with a promoter fragment deleted for *bbs* ^−1377^ was used for transformation of a strain deleted for *rbf1*, the *rbf1* deletion phenotype could not be complemented (Fig. 3D, Table 1, see below), demonstrating that *bbs* ^−1377^ is required for expression of *rbf1*. The early induction of *rbf1* upon b-activation, the presence of a conserved *bbs*-motif which is bound by the bE/bW heterodimer *in vivo*, and the requirement of this *bbs*-motif for *rbf1*-function strongly suggest that *rbf1* is a direct target of the bE/bW heterodimer.

**Table 1:** Pathogenicity of *rbf1* deletion strains.

	Strains inoculated	No. of Plants	Tumor formation (%)
Experiment 1			
	SG200	22	21 (95%)
	SG200Δ*rbf1*	74	0 (0%)
	FB1×FB2	63	59 (94%)
	FB1Δ*rbf1*×FB2Δ*rbf1*	96	0 (0%)
Experiment 2			
	SG200	20	18 (90%)
	SG200Δ*rbf1 ip::rbf1*#1	17	16 (94%)
	SG200Δ*rbf1 ip::rbf1*#2	14	12 (86%)
	SG200Δ*rbf1 ip::rbf1*#3	18	15 (83%)
	SG200Δ*rbf1 ip::rbf1Δbbs^−1377^*#1	21	0 (0%)
	SG200Δ*rbf1 ip::rbf1Δbbs^−1377^*#2	23	0 (0%)
	SG200Δ*rbf1 ip::rbf1Δbbs^−1377^*#3	22	0 (0%)

Seven-day-old maize plants were inoculated with the strains indicated and tumor formation was scored seven days post inoculation.

The cDNA-copy of *rbf1* was obtained by RACE and revealed four introns when compared to the genomic locus. The predicted open reading frame encodes a protein of 404 amino acids (aa) with an N-terminal C2H2 zinc finger domain (aa 18 to 131), a putative nuclear localization sequence (RHRR, aa 95 to 98) within the zinc finger domain and a C-terminal glutamine-rich sequence (aa 365 to 373) (Fig. 2E). To determine the localization of Rbf1, the open reading frame was fused to a triple eGFP gene and integrated into strain AB31 via homologous recombination, thereby replacing the native *rbf1* gene. Fluorescence microscopy of the resulting strain AB31*rbf1:3eGFP* (UMS63) revealed a nuclear localization of the functional Rbf1-3xGFP fusion protein upon induction of the bE/bW heterodimer (Fig. 2F), fostering the assumption that *rbf1* encodes a C2H2 zinc finger transcription factor.

### Rbf1 is required for *b*-dependent filament formation and pathogenicity

To investigate the biological function of *rbf1*, the gene was deleted in the haploid solopathogenic strain SG200 (*a1mfa2bE1bW2*) and in the haploid *U. maydis* wild-type strains FB1 (*a1b1*) and FB2 (*a2b2*), producing strains SG200Δ*rbf1* (UMS20), FB1Δ*rbf1* (UMS49) and FB2Δ*rbf1* (UMS51), respectively. In all strains, the deletion of *rbf1* did not cause any obvious phenotype in haploid sporidia growing in axenic culture, and the growth rate was not altered in different minimal or complete media (data not shown). However, when the compatible strains FB1Δ*rbf1* and FB2Δ*rbf1* were crossed on charcoal containing plates, only very short filaments were observed at the edge of the forming colonies, while the cross of FB1 or FB2 resulted in fuzzy white colonies indicative for the formation of the filamentous dikaryon (Fig. 3A). Similarly, only scarce filament formation was observed in SG200Δ*rbf1* (Fig. 3B). Since SG200 cells undergo the dimorphic switch without the need of a mating partner on charcoal containing media, we can exclude that the drastically reduced filamentation is caused by a defect in cell-cell fusion. Treatment of FB1Δ*rbf1 cells* with synthetic a2 pheromone resulted in the formation of conjugation tubes which were indistinguishable from those produced by wild-type FB1 cells, indicating that deletion of *rbf1* does not affect polarized growth *per se* (Fig. 3C). Transformation of SG200Δ*rbf1* with plasmid pRbf1 harboring the *rbf1* gene and 3kb of 5′sequence restored the fuzzy colony appearance; three independent transformants (SG200Δ*rbf1 ip::rbf1*) were indistinguishable from the SG200 wild type strain (Fig. 3 D). However, the *rbf1* deletion phenotype was not complemented when plasmid pRbf1Δbbs^−1377^, in which the *bbs*-motif at position −1377 in the *rbf1* promoter was deleted, was used for transformation (SG200Δ*rbf1 ip::rbf1Δbbs* ^−1377^) (Fig. 3 D).

To assess the role of *rbf1* during pathogenic development, seven days old maize plants were inoculated with SG200Δ*rbf1*, or with a mixture of FB1Δ*rbf1* and FB2Δ*rbf1*, and scored for tumor formation. Seven days post inoculation (dpi) 95% and 94% of the plants inoculated with SG200 and a mixture of FB1 and FB2, respectively, had developed tumors. In contrast, inoculation with the respective Δ*rbf1* mutants resulted in the complete absence of infection symptoms (Table 1). As expected, transformation of SG200*Δrbf1* with pRbf1 (SG200Δ*rbf1 ip::rbf1*) restored pathogenicity, while transformation with pRbf1Δbbs^−1377^ (SG200Δ*rbf1 ip::rbf1Δbbs^−1377^*) did not (Table 1). To determine at which stage of pathogenic development the *rbf1* mutant strains were blocked, fungal hyphae were stained with calcofluor at 2 dpi. Microscopic observation revealed that the *Δrbf1*-strains formed filaments on the leaf surface (Fig. 3E), however, we did not observe any hyphae within plant cells. To assess whether SG200Δ*rbf1* was able to form appressoria, we co-inoculated plants with a mixture of SG200 and SG200*Δrbf1* strains, each expressing either cytoplasmatically localized CFP or YFP to distinguish the strains. In the combinations SG200-CFP/SG200Δ*rbf1*-YFP and SG200-YFP/SG200Δ*rbf1*-CFP, we counted 57 and 60 appressoria for the SG200 strains on the leaf surface, respectively. By contrast, we were unable to detect any appressoria formation for the SG200*Δrbf1* strains in the same surface areas. Thus, the observed pathogenicity defect of *Δrbf1* strains results from the inability to form appressoria and to penetrate the plant cuticle.

### Rbf1 is required and sufficient to induce filamentous growth and a G2 cell-cycle arrest

**Figure 4:**
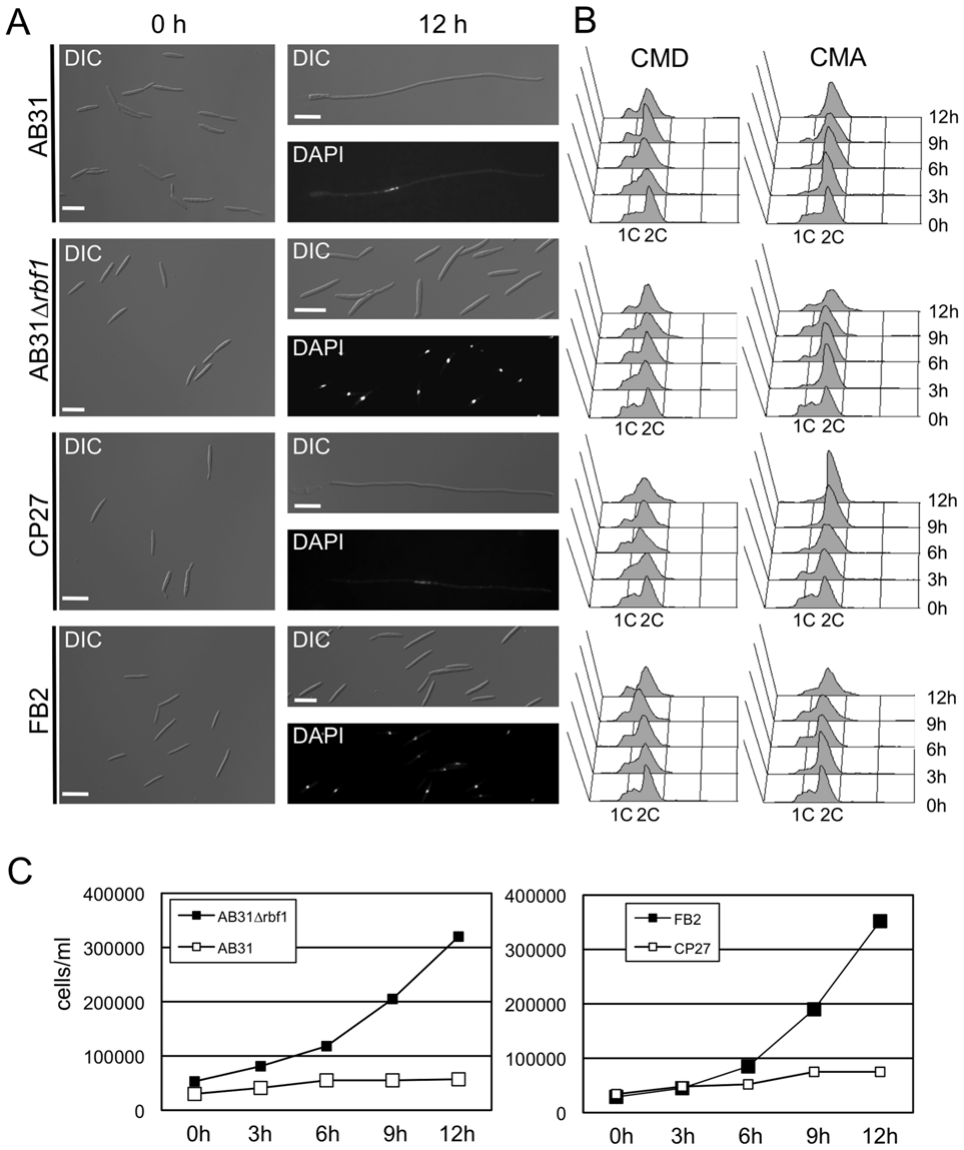
*rbf1* is required and sufficient for *b*-dependent filamentation and G2 cell cycle arrest. (*A*) Microscopic analysis of AB31, AB31Δ*rbf1*, CP27 (FB2Δ*b::P_crg1_:rbf1*) and FB2 after induction of the active bE1/bW2-heterodimer or Rbf1, respectively. Filament formation was observed 12 hours after *b*-induction in strain AB31 and *rbf1*-induction in strain CP27 (FB2Δ*b::P_crg1_:rbf1*). In contrast, strain AB31Δ*rbf1* did not switch to filamentous growth and continued to grow by budding, similar to the FB2 strain that was used as negative control. Scale bar = 10 µm. (*B*) FACS analysis of AB31, AB31Δ*rbf1*, CP27 (FB2*Δb::P_crg1_:rbf1*) and FB2 under non inducing conditions (CMD) or inducing conditions (CMA). Induction of the active *bE1/bW2* combination in AB31 or induction of *rbf1* in strain CP27 resulted in an enrichment of cells with a 2C content, indicative of a G2 cell cycle arrest. In AB31Δ*rbf1* no enrichment of cells with 2C was observed, similar to control strain FB2. (*C*) Measurement of cell numbers/ml in strains AB31, AB31Δ*rbf1*, CP27 (FB2Δ*b*::*P_crg1_:rbf1*) and FB2 grown in CMA for the time indicated. Strains AB31Δ*rbf1* and FB2 show a typical exponential growth curve, whereas strains AB31 and CP27 stopped to grow after induction of the bE1/bW2-heterodimer or *rbf1*, respectively, indicating a cell cycle arrest.

To get a more detailed view on the role of *rbf1* during *b*-dependent filament formation, we deleted the gene in strain AB31. More than 90% of the cells had switched to filamentous growth 12h after *b*-gene induction in AB31, while in AB31Δ*rbf1* (UMS25) no filament formation was observed (Fig. 4A). Upon induction of *bE1/bW2* in AB31 the cells stop to divide; in contrast, in AB31Δ*rbf1* cells continued to grow by budding (Fig. 4A and 4C), indicating that *rbf1* is required for both filamentous growth as well as for the *b*-dependent cell cycle arrest. FACS analysis of AB31 cells revealed an accumulation of cells containing a 2C DNA content upon *b*-induction, indicative for the *b*-induced G2-cell cycle arrest. In AB31*Δrbf1*, however, the distribution of cells with 1C and 2C DNA content was comparable to the wild-type strain FB2, corroborating the requirement of *rbf1* for the *b*-induced cell cycle arrest (Fig. 4B).

To dissect *b*-dependent and *rbf1*-dependent processes, we constructed strain CP27 (*a2Δb::P_crg1_:rbf1*), an FB2 derivative in which the *b*-locus was replaced by a copy of *rbf1* under control of the arabinose-inducible *crg1* promoter. Induction of *rbf1* in CP27 resulted in the formation of filamentous cells that were indistinguishable from *b*-induced filaments: the cells contained single nuclei (Fig. 4A) and stopped to divide (Fig. 4C). FACS analysis revealed that *rbf1* induction in CP27 leads to a G2 cell cycle arrest (Fig. 4B) analogous to that observed after *b*-induction. In summary, our results demonstrate that *rbf1* is required for *b*-dependent filament formation and G2 cell cycle arrest and, in addition, sufficient to induce these developmental steps in the absence of an active bE/bW-heterodimer.

### Rbf1 is a master regulator for b-dependent processes

**Figure 5:**
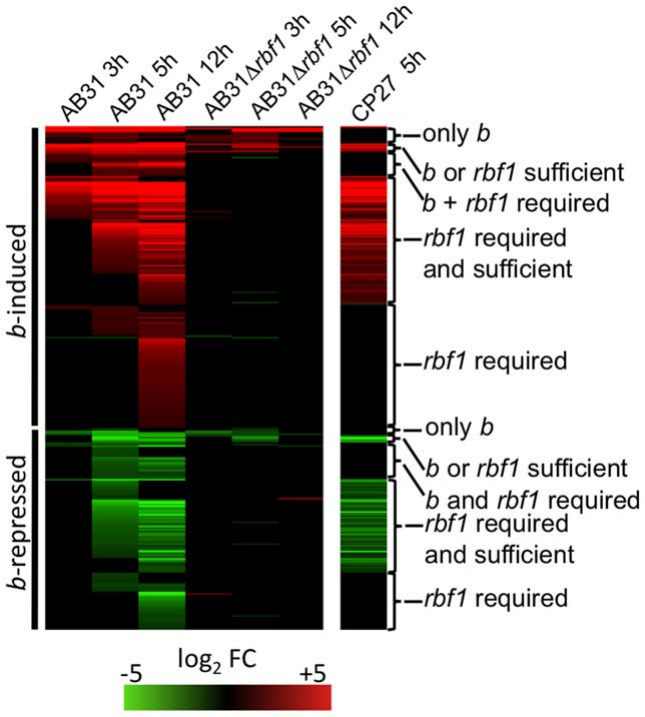
Rbf1 is required and sufficient for expression of *b*-dependent genes. The *bE1* and *bW2* genes were induced in strains AB31 or AB31Δ*rbf1*; as a control for both strains, *the* incompatible *bE2* and *bW2* genes were induced in AB32. Strain CP27 (a2 *Δb:: P_crg1_:rbf1*) was used for induced expression of *rbf1*, with strain JB2 (*a2Δb*) as control. Induction was performed by shifting the cells from array medium containing 1% glucose to array medium containing 1% arabinose. Cells were harvested at the given time points, RNA was extracted and used for microarry-analysis (Affymetrix MPIUstilagoA gene chip). The heat-map indicates the significant changes (adjusted P<0.01; change >2) in gene expression for all *b*-regulated genes depicted in Fig. 1 at the given time points relative to the control strains. Most genes do not show altered expression values in response to *b*-induction in AB31Δ*rbf1*, indicating that *rbf1* is required for the expression of the majority of *b*-dependent genes. In strain CP27, the induced expression of *rbf1* causes the induction or repression of a large group of the b-responsive genes, indicating that Rbf1 is sufficient for the regulation of these genes. Order of genes and expression changes are given in Suppl. Table S4. Details for the statistics and filters applied are given in “Material and Methods”.

To analyze the connection between *b*- and *rbf1*-mediated gene-regulation in more detail, we performed DNA-array analysis. *b*-dependent genes for which *rbf1* is required for expression were identified by comparing the transcriptional profile of strains AB31*Δrbf1* and AB31 at 3h, 5h and 12h after *b*-induction. Induced expression (5h) of *rbf1* in strain CP27 (*a2Δb::P_crg1_:rbf1*) was used to identify genes for which *rbf1* is sufficient for regulation. 189 (91.7%) out of the 206 previously identified *b*-induced genes showed no significant changes in expression after *b*-induction in strain AB31*Δrbf1* (Fig. 5; Suppl. Table S4). From the remaining 17 genes, 11 showed comparable expression levels after *b*-induction in AB31 and AB31*Δrbf1*, and 6 genes showed significant, but reduced expression levels in AB31*Δrbf1*. The 11 genes that showed no altered *b*-dependent expression upon *rbf1* deletion did not respond to *rbf1* induction in strain CP27; we consider these genes to be regulated only by *b*, and not by Rbf1 (“only *b*”, Fig. 5, Suppl. Table S4). With the exception of *um00027*, all these genes harbor sequence motifs with similarities to the *b*-binding site within their promoter sequences. In addition, they are all up-regulated early upon *b*-induction, indicating that these genes are most likely direct targets of the bE/bW heterodimer. The six genes that show a significant, but reduced *b*-responsive expression in AB31*Δrbf1* all respond to *rbf1* induction in CP27. Four of the genes harbor *b*-binding sites in their promoter regions; apparently, these genes may be regulated directly via *b* and, in addition, independently via *rbf1* (“*rbf1* OR *b* sufficient”, Fig. 5, Suppl. Table S4).

For a large fraction (46%) of the *b*-dependent genes *rbf1* is both sufficient as well as required for expression; for these genes, deletion of *rbf1* abolishes the *b*-dependent induction, and they respond to *rbf1*-induction in CP27. It is likely that the regulation of these genes occurs by a *b*-mediated regulatory cascade via Rbf1 as a central regulator (“*rbf1* required AND sufficient”, Fig. 5, Suppl. Table S4). Expression of the remaining 102 genes was dependent on *rbf1*, however, no significant induction was detected 5h after *rbf1* induction in CP27. Notably, 63 of these genes were late *b*-induced (12h after *b*-induction in AB31), and additional 22 genes were only weakly induced (less than 3-fold), or only transiently induced 5h after *b*-induction in AB31. It is well possible that these genes respond to *rbf1* only after prolonged *rbf1* induction (>5h). For 16 genes, we observed a significant *b*-dependent induction, no induction in AB31Δ*rbf1*, and no *rbf1*-dependent induction in CP27. Thus, we have to assume that for the regulation of these genes the action of both *b* and *rbf1* is required (“*rbf1* AND *b* required”, Fig. 5, Suppl. Table S4).

An analogous scenario was found for the *b*-dependently repressed genes: of the 139 *b*-dependently repressed genes, the repression was abrogated for 129 (92.8%) genes in AB31*Δrbf1*. For a total of 69 genes *rbf1* was both required and sufficient for repression. Formally, the *b*-repressed genes can be grouped equivalent to the *b*-induced genes (*b only*; *rbf1* AND *b*; *rbf1* OR *b*; only *rbf1*; Fig. 5, Suppl. Table S4).

**Figure 6:**
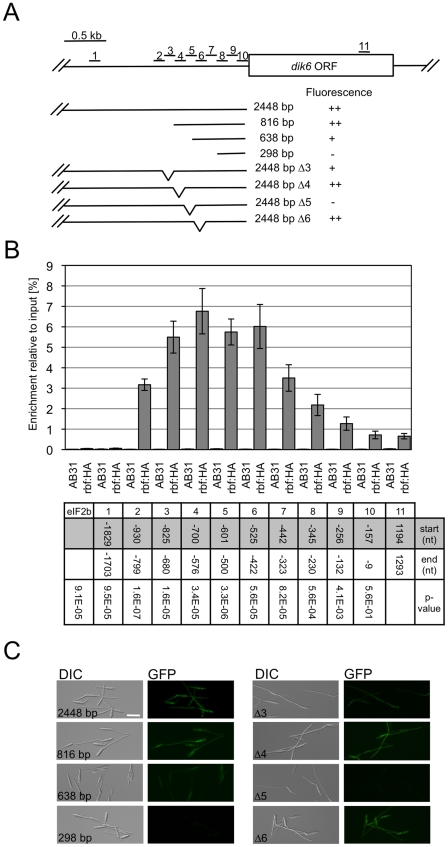
Rbf1 binds to the promoter of the Rbf1-dependently expressed *dik6* gene. (A) Overview of the *dik6* promoter region investigated by qChIP in strain AB31 *rbf1:3xHA*. Shown are the positions of the amplicons used for qChIP (numbered from 1 to 10) and the promoter truncations and internal deletions assayed in the GFP-reporter assay. (B) qChIP analysis of Rbf1-binding to the *dik6*-promoter in strains AB31 and AB31*rbf1:3xHA* 5h after induction of the bE1/bW2-heterodimer in CMA. AB31*rbf1:3xHA* expresses an HA-tagged Rbf1 protein used for immunoprecipitation with anti-HA-antibody. Numbers give the enrichment in % of the input-DNA of the PCR amplicons in DNA co-immunoprecipitated with HA-antibody Start and end of the amplicons are given in nucleotides (nt) relative to the start codon of *dik6*. The relative positions of the amplicons are given in (A). As additional control for qChIP, a region from the *eIF2b* gene (*um04869*) was used. Given are the mean values of two technical replicates of three independent experiments each, and the standard deviation (SD). Significance of the difference to values obtained for the control-amplicon located within the *dik6* open reading frame (amplicon 11) was calculated using Students t-test; the respective p-values are given for AB31*rbf1:3xHA*. Highest enrichment was observed for amplicons 3, 4, 5 and 6. No significant enrichment was observed in control strain AB31. (C) The *dik6* promoter fragments outlined in (A) were fused to GFP as a reporter and integrated in single copy into the *ip*-locus of *U. maydis* strain CP27 (*a2 Δb::P_crg1_:rbf1*). GFP-expression was visualized microscopically 5 hours after induction of *rbf1* expression in CMA medium. GFP expression declined when the promoter was truncated from 816 bp to 638 bp, and was abolished when a 298 bp promoter fragment was used. Similarly, the internal deletion Δ3 led to reduced GFP expression, while no GFP signal was detectable in the Δ 5 deletion. Scale bar = 20 µm.

To assess whether the *rbf1*-dependent gene expression involves the binding of Rbf1, we dissected the promoter of *dik6*, one of the *rbf1* responsive genes, by means of qChIP analysis. We used an AB31 derivative where the *rbf1* gene was replaced by a *rbf1*-*3xHA* fusion (AB31*rbf1:3xHA*). Induction of *bE1/bW2* in this strain triggers the expression of the Rbf1-3xHA fusion protein, which results in filamentous growth, demonstrating that the Rbf1-3xHA fusion protein is functional (Data not shown). qChIP analysis was performed via a set of 9 overlapping amplicons spanning 930 bp of the *dik6* promoter (Fig. 6A); as controls, we used an amplicon upstream of the potential promoter (−1703 to −1829 with respect to the ATG) and an amplicon within the *dik6* ORF (Fig. 6A). With the exception of an amplicon spanning the region from −9 to −157, all amplicons within the promoter showed significant (P<0.001) differences in enrichment when compared to the control amplicon located within the ORF. The amplicons with the highest enrichment were found to span the region from −825 to −422 (Fig. 6B) The functional analysis of the *dik6* promoter by means of promoter-GFP fusions revealed that Rbf1-induced GFP expression levels declined when the *dik6* promoter was truncated from 816 to 638 bp, while a 298 bp fragment was not sufficient to mediate expression (Fig. 6A,C). Internal promoter deletions corresponding to the amplicons used for the qChip analysis revealed that deletion of the *dik6* promoter region from −825 to −680 (corresponding to amplicon 3) led to reduced Rbf1-dependent induction, while the deletion of the promoter region from −601 to −500 (corresponding to amplicon 5) abolished expression completely (Fig. 6A,C). In summary, our data indicate that the *dik6* promoter has at least one binding site for Rbf1 that is required for Rbf1-mediated *dik6*-expression.

Previously, it was shown that *rbf1* is induced when haploid cells are treated with compatible pheromone [18]. Since both *bE* as well as *bW* are also induced upon pheromone treatment [19], we asked whether Rbf1 might be required for pheromone-dependent expression of the *b* genes. However, real time qRT-PCR analysis revealed no difference in the abundance of bE and bW transcripts in the strains FB1 and FB1*Δrbf1* (UMS49; *a1b1Δrbf1*) upon treatment (75 min) with synthetic a2 pheromone (see Suppl. Fig. S2). Thus, we can exclude the possibility that Rbf1 is required for the pheromone-dependent *b*-induction.

In summary, our data identify Rbf1 as the central regulatory switch within the *b*-dependent regulatory cascade, which is not only required for the regulation of the majority of the *b*-dependent genes, but also indispensable for all *b*-mediated developmental processes.

## Discussion

The switch from saprophytic to biotrophic growth of *U. maydis* requires a meticulous coordination of different processes, such as cell cycle control, the change to polarized growth, and, most interestingly, the onset of a program facilitating plant invasion and colonization. The top-most control instance for these processes is the *b*-mating type locus; it has been conclusively shown that compatible *b*-alleles are both required and sufficient for pathogenic development [5]. The bE/bW-heterodimer also controls polarized cell growth and induces a G2 cell cycle arrest, but not exclusively, since both can be triggered as well via the pheromone/receptor system encoded by the *a*-mating type locus [20]. Necessarily, the *a* and *b* loci are cross-controlled: activation of the *a*-pathway leads to induction of the *bE* and *bW* genes via Prf1, and the formation of an active bE/bW-heterodimer after cell fusion leads to a down-regulation of the *a*-pathway [19], [21].

Since a direct binding of the bE/bW heterodimer to promoters of the plethora of genes associated with *b*-dependent processes appeared unlikely, we have favored a model that places *b* on top of regulatory proteins (relays) mediating the regulation of further downstream targets. We have now identified the C2H2 zinc finger transcription factor Rbf1 as a central key player within this regulatory network.

The fast induction of *rbf1* upon b-activation, the binding of the bE/bW-heterodimer to a defined *b*-binding site in the *rbf1*-promoter region as well as the requirement of this binding site for *rbf1* function define *rbf1* as a direct target of the bE/bW-heterodimer. Deletion of *rbf1* abolishes all *b*-mediated processes, and induction of *rbf1* leads to filamentation and a G2 cell cycle arrest analogous to that observed upon *b*-induction. In addition, we could show that *rbf1* is required for regulation of the far majority of *b*-regulated genes, and, for a large fraction, also sufficient. Thus, we consider Rbf1 as a key master regulator whose action is sufficient to induce an entire complex developmental pathway. Despite of the essential function of Rbf1 within the *b*-regulatory cascade, we consider it unlikely that *rbf1* alone is sufficient to trigger pathogenic development of *U. maydis*, because *clp1*, which was shown to be required for pathogenicity [8], is induced directly by *b* and independently from *rbf1*. We were not able to address this question experimentally, since transformants with a constitutively expressed *rbf1* gene were not viable, most probably as a result of the *rbf1* induced cell cycle arrest.

In fungi, only few master regulators of pathogenic development have been identified yet. In *Candida albicans*, WOR1 is the master regulator of white to opaque switching [22], and the *C. neoformans* Gat201 [23] is a key regulator of melanin production and capsule formation. The *C. neoforman*s Cir1 transcriptional regulator integrates the sensing of iron with the expression of virulence factors, with signalling pathways influencing virulence, and with growth at elevated temperature [24], [25]. WOR1 and Gat201 are required (and sufficient) for the initiation of specific programs that are tightly linked to fungal pathogenesis. In contrast, nearly all of the genes regulated by *b* require *rbf1* for their expression, and it is not possible to assign specific, common functions to the *rbf1*-regulated genes, or to the few genes that are not regulated by Rbf1. Thus, different from WOR1 and Gat201, Rbf1 regulates not the genes of a specific, defined pathway, but is required for the regulation of all *b*-dependent processes. Similarly, the *C. neoformans* Cir1 regulator is involved in the regulation of all major virulence traits [24], [25].

The cell cycle block of the *b*-induced filaments is only released upon plant penetration. Our data reveal a complex contribution of different key players to control the cell cycle. At least four different transcription factors, namely bE/bW, Rbf1, and the two Rbf1-dependent factors Biz1 and Hdp1 are involved in cell cycle regulation. The ectopic expression of any of these factors leads to the formation of G2 arrested hyphae [6], [8, C. Pothiratana and J. Kämper, unpublished], which argues for a complex transcriptional network with different levels of relays that allow the integration of various stimuli, as for example, the unknown signal that leads to the release of the cell cycle after penetration of the host plant. The regulatory control achieved via b, Rbf1, Biz1 and Hdp1 may funnel into the transcriptional regulation of different key factors for cell cycle control, as we observe the transcriptional regulation of different cyclins (*cln1*, *clb1*, *clb2 and pcl12*) and of the potential Wee1 kinase Um03928. The Um03928 homologue in *S. pombe*, Cdr2, is required for the proper formation of septae, and functions as mitotic inducer via the negative regulation of the central cell cycle regulator Wee1 [15], [26]; the *U. maydis* Wee1 was shown to trigger filamentous growth and a G2 arrest [9], [16]. Obviously, the *b*-induced G2 cell cycle arrest is controlled by several independent regulatory pathways.

The induction of *b* leads to the formation of polar growing hyphae, and several of the *b*-dependently regulated genes reflect this morphological change and the altered requirements of the cell for e.g. long distance transport or cell wall remodeling. However, the most interesting trait by which the *b*-induced filament differs from other filaments like the pheromone-induced conjugation tube is its ability to infect the host. The exploitation of the *b*-dependently regulated genes provides for the first time comprehensive insights into the complex developmental processes during morphogenic switching and pathogenic development of *U. maydis*. The pathogenic potential of the hyphae may for once be marked by an altered cell wall composition, as we observe the differential regulation of several genes involved in cell wall synthesis, including chitin synthases and chitin deacetylases. Rebuilding or masking of the cell wall is a strategy of pathogens to evade perception or to protect themselves from defense responses of the host [27]. However, deletion of either of the two *b*-regulated chitin deacetyases does not affect virulence in *U. maydis* (B. Günther, J. Kämper, B. Moerschbacher, unpublished), and neither are the two chitin synthases *chs1* and *chs4* required for pathogenicity [28], most likely due to overlapping and/or redundant functions.

The other intriguing characteristic of the *b*-filament is the secretion of various potential effector proteins. Such effectors are thought to be involved in suppression of host defense responses and redirection of nutrient flow during biotrophic growth. The expression of putative effectors prior to the contact with the plant indicates a priming mechanism of the fungal hypha to facilitate rapid suppression of plant defense responses and the fast establishment of the biotrophic interface subsequent to plant penetration. The observation that the temperature-induced inactivation of the bE1/bW2-heterodimer *in planta* abolishes expression of various additional candidate effector genes [29] that are not identified as *b*-regulated in this study, implies that the temporal expression of these genes is subject to combinatorial gene regulation involving the bE/bW-heterodimer and other plant-induced factors.

The competence of the *b*-filaments to penetrate the plant cuticula is reflected by the induction of Biz1 and the MAP kinase Kpp6. Both factors have been shown to be required for efficient formation of appressoria and subsequent penetration. Rbf1 is required for the *b*-dependent induction of both genes, which explains the absence of appressoria in *rbf1* mutant strains.

Rbf1 is required for the induction of most, but not all *b*-regulated genes. All genes that are exclusively regulated by bE/bW harbor b-binding sites within their promoters, and it is conceivable that these genes are regulated via direct binding of the bE/bW-heterodimer. The majority of the *b*-regulated genes, however, lack putative *b*-binding sites, indicating that the *b*-dependent regulatory circuit involves additional transcription factors.

Similarly, those genes for which *rbf1* is required and sufficient for regulation may be directly regulated by Rbf1. Our data indicate that Rbf1 binding to the promoter of the dik6 gene is required for Rbf1-mediated *dik6* expression, which emphasizes the function of Rbf1 as a transcription factor. However, the actual Rbf1 binding site has not been determined yet. The *in silico* analysis of *rbf1*-regulated genes may be constrained by the fact that Rbf1 triggers the induction of at least three transcription factors, leading to a superimposition of direct and indirect effects. For a small fraction of genes, both *b* and *rbf1* are required for regulation, which can be explained by a combinatorial action of two transcription factors [30]. A substantial number of genes is down-regulated upon *b*-induction. Intriguingly, two of the very few known transcription factors that can act both as transcriptional activators and repressors, the *S. cerevisiae* Rme1 protein and the human YY1 protein, are both C2H2 zinc-finger proteins [31], [32]. Thus, it is well possible that the repression of genes is also directly mediated via Rbf1.

**Figure 7:**
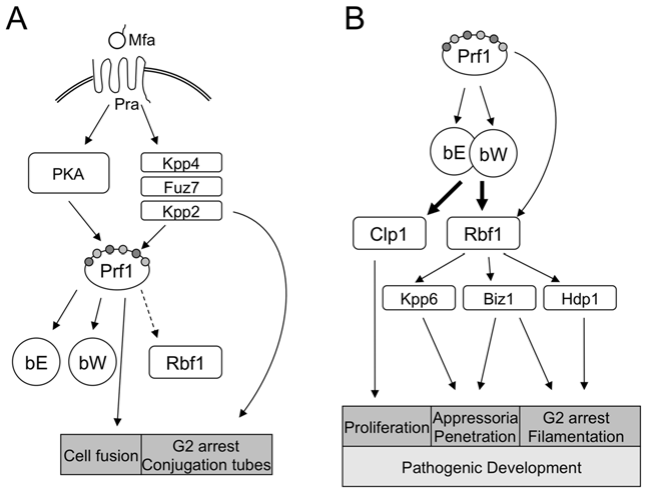
Key factors of *a*- and *b*-dependent development in *U. maydis*. (*A*) Activation of the pheromone pathway after pheromone (Mfa) binding to the cognate receptor (Pra) triggers a signalling casacde via PKA and MAPK phospho-relays. Differential phosphorylation of the HMG transcription factor Prf1, in turn, leads to expression of the *b*-mating type locus gene bE and bW. (*B*) After cell fusion, sexual and pathogenic development is orchestrated by the bE/bW-heterodimer, coordinating the key players of important developmental steps: Clp1 is required for *in planta* proliferation; Rbf1 is the central transcriptional regulator, controlling appressoria formation and penetration of the host plant via the transcription factor Biz1 and the MAPK Kpp6, as well as filamentous growth and the G2 cell cycle arrest by concerted action of Biz1 and Hdp1.

The dimorphic switch and the onset of pathogenic development trigger a multilayered regulatory cascade that involves several transcription factors (Fig. 7). Is there a specific reason that the bE/bW-heterodimer regulates only a small number of genes directly and more than 90% in dependence on a second master regulator? For once, additional regulators allow more signals to be integrated into the regulatory circuits, which may help to quickly adapt to changing environmental conditions during biotrophic development, thereby avoiding nutrient stress or plant defense responses. In particular, Rbf1 interconnects the *a*- and *b*-dependent regulatory pathways, as both pheromone-response [18] as well as *b*-induction leads to *rbf1* expression. Since we could not determine a specific function for Rbf1 in the pheromone-dependent signalling pathway and deletion of *rbf1* is not required for conjugation tube formation and for pheromone-induced G2 cell cycle arrest, we consider it unlikely that *rbf1* plays a central role in *a*-dependent signalling or gene regulation. One possibility is that the pheromone-induced expression of *b* and *rbf1* primes the cells for post-fusion events. The *a*- and *b*-dependently induced cell cycle arrest is independently coordinated; thus, the pheromone-induced *rbf1* expression facilitates rapid switching of developmental programs thereby minimizing the time preceding plant infection (Fig. 7).

In essence, our study provides fundamental new insights into the complex regulatory traits of sexual, as well as pathogenic development of *U. maydis*. The identification of key factors points towards an emerging picture that explains how multilayered regulatory pathways can dynamically interact to control complex developmental decisions. We believe that this work is not only relevant for *U. maydis* but can also serve as a model for other fungi and higher organisms.

## Methods

### Strains and growth conditions

*Escherichia coli* strain TOP10 (Invitrogen) was used for cloning purposes. Growth conditions and media for *E. coli* [33] and *U. maydis* [8], [34], [35] and the quantification of appressoria formation [6] have been described previously. *U. maydis* strains with relevance for this study are listed in Suppl. Table S5. *U. maydis* strains carrying *crg1* expression constructs were induced in array medium [8] or CM medium [34] supplemented with 1% arabinose instead of 1% glucose as described in [8]; equivalently, for *nar1*-induction, array medium supplemented with 3.8g/l KNO_3_ instead of glutamine (4.38 g/l) as nitrogen source was used. Mating assays and plant infections are described in reference [35]. For pheromone stimulation of *U. maydis* cells we followed the protocol of [36].

### DNA and RNA procedures

Molecular methods followed described protocols [33]. DNA isolation and transformation procedures for *U. maydis* were carried out as described [37]. For all gene deletions, we used the PCR based approach described in [38]. For the Rbf1-3xeGFP fusion, 1 kb of the 3′end of the *rbf1* ORF and 1 kb of the 3′ UTR were PCR-amplified, introducing two *Sfi*I sites and removing the stop-codon of *rbf1*; both fragments were ligated to an *Sfi*I 3xeGFP-Hyg^R^ fragment of pUMA647 (K. Zarnack and M. Feldbrügge, unpublished) in pCR2.1 TOPO (Invitrogen) as backbone, yielding pMS85.

To replace the *b*-mating type locus with the arabinose inducible *rbf1* allele, the *rbf1*-ORF was PCR amplified, creating an *Nde*I site at the start and a *Not*I site following the stop codon, and cloned into pCR2.1 TOPO. The *Nde*I-*Not*I *rbf1*-ORF-fragment, a 1.3 kb *BstX*I(blunt)-*Nde*I *crg1*-promotor fragment and a 0.3 kb *Not*I-*EcoR*I(blunt) nos-terminator fragment from pRU12 [11] were integrated into the *Stu*I site of pCRΔb [38] to generate pCRΔb-crg:rbf1. For the generation of HA-tagged bE1- and Rbf1-fusion proteins 1 kb of the 3′end of the ORF and 1 kb of the 3′ UTR were PCR-amplified, introducing two *Sfi*I sites and removing the stop-codon; respective fragments were ligated to an *Sfi*I 3xHA-Hyg^R^ fragment of pUMA792 (M. Feldbrügge, unpublished) and cloned into pCRII TOPO, yielding plasmids pDS1 and pDS3. After linearization plasmids were integrated into the *bE1* and *rbf1* loci, respectively, of strain AB31 by homologous recombination.

All PCR amplified fragments were verified by sequencing. For transformation, either linearized plasmid DNA or PCR generated linear DNA was used; homologous integration was verified by Southern blot. For complementation of the *rbf1* deletion, a 3kb region upstream of the *rbf1* ORF was PCR amplified introducing a 5′-*Fse*I and a 3′-*Nde*I restriction site and inserted with the *Nde*I-*Not*I *rbf1*-ORF fragment of pCRΔb-crg:rbf1 into pRU11-*Not*I6474 (a pRU11 [11] derivative in which the *Not*I site at position 6474 has been removed by a fill up reaction) by three-fragment ligation to generate pRbf1. Generation of pRbf1Δbbs^−1377^ was performed as described for pRbf1, with the exception that the *bbs*-motif at position −1377 within the 3kb *rbf1*- upstream region was removed by standard PCR techniques [39].

For generation of *dik6* promoter-GFP fusion constructs the 2448 bp *dik6* promoter fragment was PCR-amplified and integrated into pRU4 [11] digested with *Hpa*I and *Nde*I. From the resulting plasmid *dik6* promoter fragments of 816 bp, 638 bp and 298 bp were recovered as *Bcl*I(blunt)/*Nde*I, *Msc*I(blunt)/*Nde*I and *Hind*III(blunt)/*Nde*I fragments and integrated into pRU4 [11] digested with *Hpa*I and *Nde*I. Internal deletions in the dik6 promoter were introduced by standard PCR techniques [39]. PCR amplified fragments were integrated into pRU11 via *Fse*I/*Nde*I restriction sites [11].

RNA extraction and qRT-PCR analysis for *rbf1*, *bW*, *bE* and *ppi* was performed as described [8]. Full-length *rbf1* cDNA was isolated using the GeneRacer Kit (Invitrogen), cloned in pCR2.1 TOPO (Invitrogen) and sequenced. For overview of primers used see Suppl. Table S6.

### Quantitative Chromatin Immunoprecipitation (qChIP)

50 ml cultures of *U. maydis* were grown until OD_600_ = 0,6–1,0 and cross-linked by addition of formaldehyde (1% final concentration) for 15 min at RT; glycine was added to a final concentration of 0.125 M, cells were harvested by centrifugation and washed three times in TBS (50 mM Tris-HCl, 150 mM NaCl, pH 7.6). The pellet was resupended in 1.5 ml FA lysis buffer (50 mM HEPES-KOH [pH 7.5], 150 mM NaCl, 1 mM EDTA, 1% [v/v] Triton-X-100, 0.1% [w/v] sodium deoxycholate, 0.1% [w/v] sodium dodecyl sulfate [SDS]) supplemented with 2 mM PMSF, 5 mM benzamidine and 1× Complete EDTA-free (Roche). Cells were lysed with a cell mill (Retsch MM200, 25Hz, 5min) in liquid nitrogen pre-cooled grinding jars and the powdery cell extract thawed on ice. 1 ml aliquots of the resulting suspension were sonicated on ice; sonication was set to yield a DNA average size of 400–500 bp. After centrifugation (17000g, 15 min, 4°C) the supernatant was used as the input sample in immunoprecipitation experiments. For each experiment, 400 µl aliquots of the input sample were incubated with 25 µl monoclonal anti-HA-agarose beads (Sigma-Aldrich) over night at 4°C on a rotating wheel.

Washing of beads and recovery of the immunoprecipitated DNA was done according to the ChIP protocol from the Haber Lab (http://www.bio.brandeis.edu/haberlab/jehsite/protocol.html) with the following modifications. All washing steps were carried out at 4°C and repeated one more time, with exception of the TE wash. Proteinase K treatment was done with 50 µl TE containing 3.5 mg/ml Proteinase K without glycogen, and phenol/chlorophorm extraction was done without LiCl. Samples were analysed by qPCR on a Bio-Rad iCycler using the Mesa Green qPCR MasterMix Plus (Eurogentec) with 400 nM Primer (each) and 1 µl immunoprecipitated DNA or 1/100 diluted input DNA, respectively. Amplicons were normalized to input DNA using the Bio-Rad IQ5 software.

### DNA array and data analysis

Custom-designed Affymetrix chips were used for DNA-array analysis. Probe sets for the individual genes are visualized at http://mips.helmholtz-muenchen.de/genre/proj/ustilago/Target preparation, hybridization and data analysis was performed essentially as described before [40], with the following alterations: 5 µg RNA were used for first strand cDNA synthesis at 50°C with Superscript II (Invitrogen); for all experiments, an adjusted P-value for the false discovery rate [41] of ≤0.01 and a change in expression of ≥2 was used for filtering. For analysis of *b*-dependent gene expression strain AB31 (*a2 P_crg1_:bE1/bW2*) was compared to strain AB32 (*a2 P_crg1_:bE2/bW2*) and strain AB33 (*a2 P_nar1_:bE1/bW2*) was compared to strain AB34 (*a2 P_nar1_:bE2/bW2*) at the given time points. For analysis of *rbf1*-dependent gene expression strain AB31 (*a2 P_crg1_:bE1/bW2*) was compared to AB31Δ*rbf1* (*a2 P_crg1_:bE1/bW2* Δ*rbf1*) and strain CP27 (*a2 Δb::P_crg1_:rbf1*) was compared to strain JB2 (*a2 Δb*) at the given time points. Expression values were calculated as mean of two biological replicates. All array data have been submitted to GEO/NCBI (GSE18750, GSE18754 and GSE18756).

*De novo* promoter motif search was performed using the TAMO framework [42] extended to include AlignAce, Bioprospector, Cismodul, Improbizer, Meme, MDScan and Weeder. Output of each algorithm was collected, converted into a position weight matrix and scored with a hypergeometric test reflecting a random selection null hypothesis [43].

### FACS analysis

Flow cytometry measurements were performed as described before [20]. Cell counting was performed with a Neubauer counting chamber.

### Microscopy

Microscopic analysis was performed using an Axioimager equipped with an Axiocam MRm camera or a Lumar V12 equipped with an Axiocam HRc (Zeiss, Jena, Germany). Nuclei were stained with DAPI Vectashield H-1200 (Vector Laboratories), fungal cell walls with 2 µg/ml Calcofluor white (Sigma, St. Louis, MO) in PBS. All images were processed with Axiovision (Zeiss, Jena, Germany).

### Accession numbers

*clp1* (um02438) XP_758585, *rbf1* (um03172) XP_759319, *hdp1* (um12024) XP_761909.1, *hdp2* (um04928) XP_761075, *biz1* (um02549) XP_758696, *cln1* (um04791) XP_760938, *clb1* (um03758) XP_759905, *clb2* (um10279) XP_758735, cdr2-like protein (um03928) XP_760075, *pcl12* (um10529.2) XP_760585, DNA polymerase epsilon (um01008) XP_757155, DNA replication licensing factor (um06402) XP_762549.

## Supporting Information

Figure S1 Kinetics of bW- and bE-expression driven by the arabinose inducible *crg1*-promoter (strains AB31, AB32) and the nitrate inducible *nar1*-promoter (strains AB33, AB34) in *U. maydis*.
(3.75 MB TIF)

Figure S2 Rbf1 is not required for pheromone-induced *b*-expression.
(1.04 MB TIF)

Table S1 Alteration of gene expression in response to induced expression of bE1/bW2.
(0.27 MB XLS)

Table S2 Overview of genes previously published as *b*-regulated.
(0.03 MB XLS)

Table S3 Enrichment analysis for GO-categories of *b*-regulated genes.
(0.11 MB XLS)

Table S4 Overview of gene expression after deletion/induction of *rbf1*.
(0.16 MB XLS)

Table S5 *U. maydis* strains used in this study.
(0.04 MB DOC)

Table S6 Primer list.
(0.02 MB XLS)
