# Supplementary figures and images for: The Transcription Factor Rbf1 Is the Master Regulator for b-Mating Type Controlled Pathogenic Development in Ustilago maydis
Source: PLoS Pathog. 2010 Aug 5;6(8):e1001035. doi: 10.1371/journal.ppat.1001035 (PMC2916880; doi:10.1371/journal.ppat.1001035)

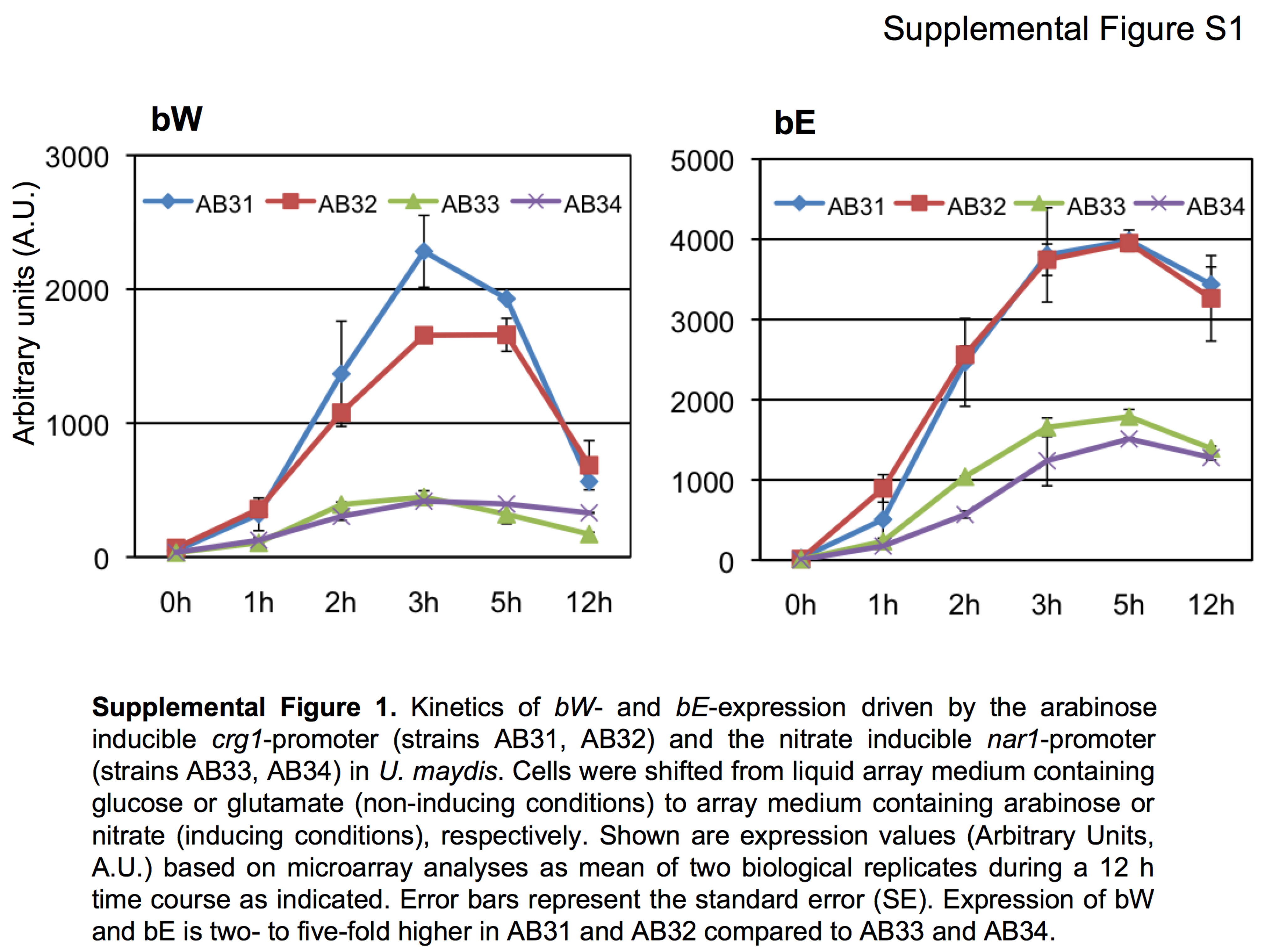

Supplement: Figure S1 — Kinetics of bW- and bE-expression driven by the arabinose inducible crg1-promoter (strains AB31, AB32) and the nitrate inducible nar1-promoter (strains AB33, AB34) in U. maydis. (3.75 MB TIF) [file ppat.1001035.s001.tif]

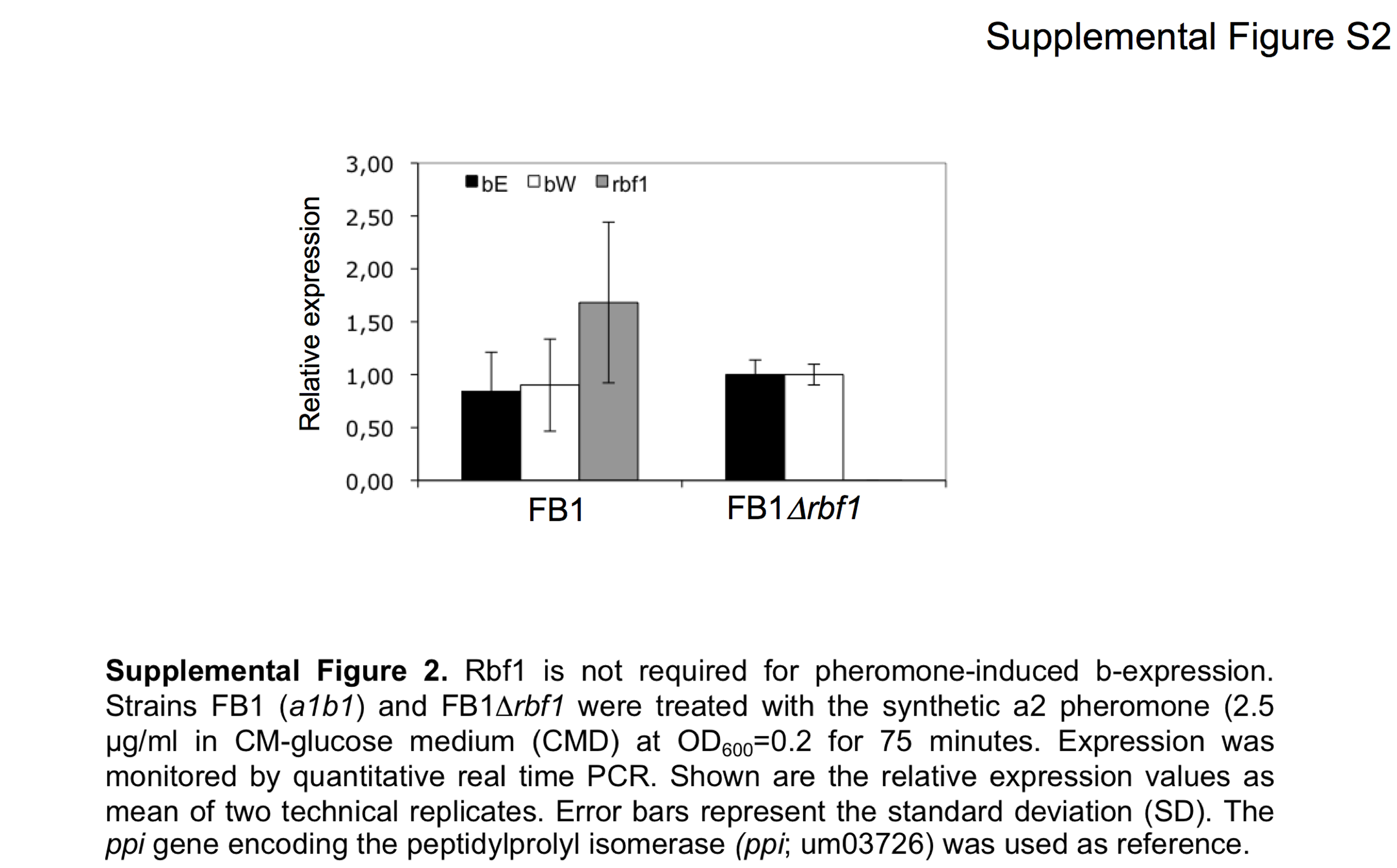

Supplement: Figure S2 — Rbf1 is not required for pheromone-induced b-expression. (1.04 MB TIF) [file ppat.1001035.s002.tif]
