# Supplementary material for: The Transcription Factor Rbf1 Is the Master Regulator for b-Mating Type Controlled Pathogenic Development in Ustilago maydis
Source: PLoS Pathog. 2010 Aug 5;6(8):e1001035. doi: 10.1371/journal.ppat.1001035 (PMC2916880; doi:10.1371/journal.ppat.1001035)
Supplement: Table S5 — U. maydis strains used in this study. (0.04 MB DOC) [file ppat.1001035.s007.doc]

**Supplemental Table S5.** *U. maydis* strains used in this study

**Strain Relevant Genotype Reference**

AB31 *a2* *P_crg_:bW2,bE1* Brachmann et al. (2001)

AB32 *a2* *P_crg_:bW2,bE2* Brachmann et al. (2001)

AB33 *a2* *P_nar_:bW2,bE1* Brachmann et al. (2001)

AB34 *a2* *P_nar_:bW2,bE2* Brachmann et al. (2001)

FB1 *a1 b1* Banuett and Herskowitz (1989)

FB2 *a2 b2* Banuett and Herskowitz (1989)

SG200 *a1mfa2 bW2bE1* Kämper et al. (2006)

JB2 *a2 Δb* Wahl et al. (2010)

SG200*Δb a1mfa2 Δb* Kämper (2004)

SG200CFP *a1mfa2 bW2bE1 ip^r^*[*P_oma_:cfp*]*ip^s^* Flor-Parra et al. (2006)

SG200YFP *a1mfa2 bW2bE1 ip^r^*[*P_oma_:yfp*]*ip^s^* Flor-Parra et al. (2006)

UMS25 *a2* *P_crg_:bW2,bE1* *Δrbf1* This study

UMS63 *a2* *P_crg_:bW2,bE1 rbf1:3eGFP* This study

CP27 *a2* *Δb::P_crg_:rbf1* This study

UMS20 *a1mfa2 bW2bE1 Δrbf1* This study

UMS49 *a1 b1 Δrbf1* This study

UMS51 *a2 b2 Δrbf1* This study

UMS87 *a1mfa2 bW2bE1Δrbf1 ip^r^*[*P_oma_:cfp*]*ip^s^* This study

UMS88 *a1mfa2 bW2bE1Δrbf1 ip^r^*[*P_oma_:yfp*]*ip^s^* This study

AB31*bE1:3xHA* *a2* *P_crg_:bW2,bE1:3xHA* This study

AB31*rbf1:3xHA* *a2* *P_crg_:bW2,bE1 rbf1:3xHA* This study

SG200Δ*rbf1 a1mfa2 bW2bE1 Δrbf1 ip::rbf1 +bbs* This study

*ip::rbf1*

SG200Δ*rbf1 a1mfa2 bW2bE1 Δrbf1 ip::rbf1 -bbs* This study

*ip::rbf1Δbbs-1377*

CP27 *a2* *Δb::P_crg_:rbf1 ip^r^*[*Pdik6_2448:gfp*]*ip^s^* This study

Pdik6_2448_:GFP

CP27 *a2* *Δb::P_crg_:rbf1 ip^r^*[*Pdik6_816:gfp*]*ip^s^* This study

Pdik6_816_:GFP

CP27 *a2* *Δb::P_crg_:rbf1 ip^r^*[*Pdik6_638:gfp*]*ip^s^* This study

Pdik6_638_:GFP

CP27 *a2* *Δb::P_crg_:rbf1 ip^r^*[*Pdik6_298:gfp*]*ip^s^* This study

Pdik6_298_:GFP

CP27 *a2* *Δb::P_crg_:rbf1 ip^r^*[*Pdik6_2448∆3:gfp*]*ip^s^* This study

Pdik6_2448_:GFP∆3

CP27 *a2* *Δb::P_crg_:rbf1 ip^r^*[*Pdik6_2448∆4:gfp*]*ip^s^* This study

Pdik6_2448_:GFP∆4

CP27 *a2* *Δb::P_crg_:rbf1 ip^r^*[*Pdik6_2448∆5:gfp*]*ip^s^* This study

Pdik6_2448_:GFP∆5

CP27 *a2* *Δb::P_crg_:rbf1 ip^r^*[*Pdik6_2448∆6:gfp*]*ip^s^* This study

Pdik6_2448_:GFP∆6

**References:**

Banuett, F. and Herskowitz, I. 1989. Different *a* alleles of *Ustilago maydis* are necessary for maintenance of filamentous growth but not for meiosis. *Proc Natl Acad Sci USA* **86**: 5878-5882.

Brachmann, A., Weinzierl, G., Kämper, J., and Kahmann, R. 2001. Identification of genes in the bW/bE regulatory cascade in *Ustilago maydis*. *Mol Microbiol* **42**(4): 1047-1063.

Flor-Parra, I., Vranes, M., Kämper, J., and Perez-Martin, J. 2006. Biz1, a zinc finger protein required for plant invasion by *Ustilago maydis,* regulates the levels of a mitotic cyclin. *Plant Cell* **18**(9): 2369-2387.

Kämper, J. 2004. A PCR-based system for highly efficient generation of gene replacement mutants in *Ustilago maydis*. *Mol Genet Genomics* **271**(1): 103-110.

Kämper, J., Kahmann, R., Bölker, M. *et. al.* 2006. Insights from the genome of the biotrophic fungal plant pathogen *Ustilago maydis*. *Nature* **444**(7115): 97-101.

Wahl,R., Zahiri, A., and Kämper, J., 2010. The Ustilago maydis b mating type locus controls hyphal proliferation and expression of secreted virulence factors in planta. *Mol Microbiol* **75**(1):208-20.
